# FEZF1-AS1 functions as an oncogenic lncRNA in retinoblastoma

**DOI:** 10.1042/BSR20190754

**Published:** 2019-05-31

**Authors:** Lian-Jiao Quan, Wen-Jun Wang

**Affiliations:** 1 Department of Ophthalmology, Qingyang People’s Hospital, Qingyan, Gansu 745000, China; 2 Department of Ophthalmology, Xi’an No.4 Hospital, Xi’an, Shaanxi 710004, China; 3 Department of Ophthalmology, Shaanxi Ophthalmic Medical Center, Xi’an, Shaanxi 710004, China; 4 Department of Ophthalmology, Affiliated Guangren Hospital, School of Medicine, Xi’an Jiaotong University, Xi’an, Shaanxi 710004, China

**Keywords:** biomarkers, cancer, FEZF1-AS1, large intervening non-coding RNA, retinoblastoma

## Abstract

Long non-coding RNA (lncRNA) FEZF1 antisense RNA 1 (FEZF1-AS1) has been shown to be up-regulated in tumor tissues and cells, and exerts oncogenic effects on various types of malignancies. However, the expression and function of FEZF1-AS1 was still fully unclear in retinoblastoma. The purpose of our study was to investigate the expression and clinical value of FEZF1-AS1 in retinoblastoma patients, and explore the effect of FEZF1-AS1 on retinoblastoma cell proliferation, migration and invasion. In our results, levels of FEZF1-AS1 expression were elevated in retinoblastoma tissue specimens and cell lines compared with adjacent normal retina tissue specimens and human retinal pigment epithelial cell line, respectively. The correlation analysis indicated that high FEZF1-AS1 expression was significantly correlated with present choroidal invasion and optic nerve invasion. Survival analysis suggested that retinoblastoma patients in high FEZF1-AS1 expression group had obviously short disease-free survival (DFS) compared with retinoblastoma patients in low FEZF1-AS1 expression group, and high FEZF1-AS1 expression was an independent unfavorable prognostic factor for DFS in retinoblastoma patients. Loss-of-function study indicated silencing FEZF1-AS1 expression inhibited retinoblastoma cell proliferation, invasion and migration. In conclusion, FEZF1-AS1 functions as an oncogenic lncRNA in retinoblastoma.

## Introduction

Retinoblastoma originated from the retinal photoreceptor precursor cells, and is the most common childhood intraocular cancer accounting for 3% of all pediatric cancers [1,2]. The incidence of retinoblastoma is about 1 case in every 15000–20000 live births [3]. Although the survival rate of retinoblastoma patients is up to 95% in developed countries, the survival rate of patients with retinoblastoma is still less than 70% in developing countries and less developed countries [4,5]. The tumorigenesis of retinoblastoma is a highly complicated process, which has close relationship with gene mutation [6,7], oncogene aberrant expression [8,9], and activation of oncogenic signaling pathways [10,11]. The molecular mechanisms underlying retinoblastoma occurrence and development remain unknown. Therefore, it is very useful to investigate the molecular mechanisms of retinoblastoma initiation to develop new targeted therapy and improving clinical outcome.

Long non-coding RNAs (lncRNAs) are identified as RNA transcripts greater than 200 nts in length and limited protein-coding potential [12]. More and more reports have suggested that lncRNAs play key roles in the development and progression of retinoblastoma, such as PlncRNA-1 [13], ANRIL [14], THOR [15], AFAP1-AS1 [16], XIST [17] and so on. lncRNA FEZF1 antisense RNA 1 (FEZF1-AS1) is localized at the human chromosome 7q31.32, and is transcribed in the opposite direction of *FEZF1* gene. FEZF1-AS1 has been shown to be up-regulated in tumor tissues and cells, and exert oncogenic effects on lung cancer [18–21], breast cancer [22], liver cancer [23,24], gastric cancer [25–27], colorectal cancer [28,29], pancreatic cancer [30,31], ovarian cancer [32], cervical cancer [33], osteosarcoma [34], nasopharyngeal carcinoma [35] and multiple myeloma [36]. However, the expression and function of FEZF1-AS1 was still fully unclear in retinoblastoma. The purpose of our study was to investigate the expression and clinical value of FEZF1-AS1 in retinoblastoma patients, and explore the effect of FEZF1-AS1 on retinoblastoma cell proliferation, migration and invasion.

## Materials and methods

### Clinical specimens

Total 60 fresh retinoblastoma tissue specimens and 30 adjacent normal retina tissue specimens were obtained from retinoblastoma patients who did not receive any preoperative anti-tumor treatment and underwent surgery at Qingyang People’s Hospital, Xi’an No.4 Hospital, Shaanxi Ophthalmic Medical Center or Affiliated Guangren Hospital School of Medicine Xi’an Jiaotong University. Adjacent normal retina tissues were at least 1 cm away from the edge of the primary tumor with no obvious tumor cells. All clinical specimens were immediately frozen in liquid nitrogen after surgery, and pathologically confirmed by at least two pathologists. This research was conducted with the approval of the Ethics Review Committee of Qingyang People’s Hospital, Xi’an No.4 Hospital, Shaanxi Ophthalmic Medical Center and Affiliated Guangren Hospital School of Medicine Xi’an Jiaotong University. The written informed consents were gained from patients or their guardians.

### RNA isolation and qRT-PCR

Total RNAs were isolated from tissue specimens or cell lines by using TRIzol reagent (Invitrogen, Carlsbad, CA, U.S.A.), and were used for template to reverse-transcribe into cDNAs by using PrimeScript RT reagent Kit (Takara Biomedical Technology, Beijing, China). The qRT-PCR was performed through utilizing One Step TB Green PrimeScript RT-PCR Kit II (Takara Biomedical Technology, Beijing, China) according to the manufacturer’s protocols. The specific primers were: FEZF1-AS1, forward, 5′-AGAGGCTATGACTCAGGGTT-3′ and reverse, 5′-TGTTGCTCCACAGTAAAGGT-3′; GAPDH, forward, 5′-GCACCGTCAAGGCTGAGAAC-3′ and reverse, 5′-TGGTGAAGACGCCAGTGGA-3′. Relative FEZF1-AS1 expression was normalized to GAPDH.

### Cell lines and cell transfection

Human retinoblastoma cell lines (Weri-RB-1, Y79 and RBL-13) were grown in Roswell Park Memorial Institute (RPMI)-1640 medium (HyClone, Logan, UT, U.S.A.) supplemented with 10% fetal bovine serum (FBS, HyClone, Logan, UT, U.S.A.). Human retinal pigment epithelial cell line (ARPE-19) was grown in Dulbecco’s modified Eagle’s medium (DMEM) containing 10% FBS (HyClone, Logan, UT, U.S.A.). All cells were maintained in a humidified incubator with 5% CO_2_ at 37°C. To silence FEZF1-AS1 expression, three individual siRNAs (FEZF1-AS1-siRNA#1, FEZF1-AS1-siRNA#2, and FEZF1-AS1-siRNA#3) and an extra negative control siRNA (NC-siRNA) were designed and optimized by GenePharma Corporation. The sequences of siRNA-FEZF1-AS1s were shown as follows: FEZF1-AS1-siRNA#1 (sense, 5′-AAACAUGGCAGCUACAAGACGGGUC-3′ and antisense, 5′-GACCCGUCUUGUAGCUGCCAUGUUU-3′); FEZF1-AS1-siRNA#2 (sense, 5′-CAGGUACCACAAAGCCACUAGUGCA-3′ and antisense, 5′-UGCACUAGUGGCUUUGUGGUACCUG-3′); FEZF1-AS1-siRNA#3 (sense, 5′-CCAGGACUGGGCAGUGCAUUCUUUA-3′ and antisense, 5′-UAAAGAAUGCACUGCCCAGUCCUGG-3′). The siRNAs were transfected into retinoblastoma cells through using Lipofectamine 3000 reagent (Invitrogen, Carlsbad, CA, U.S.A.) according to the manufacturer’s protocols. The qRT-PCR was utilized to confirm the transfection efficiencies of FEZF1-AS1-siRNAs in retinoblastoma cells.

### Cell counting kit-8 assay

Cell counting kit-8 (CCK-8) assay was used to estimate the cell proliferation ability of retinoblastoma cells. Briefly, transfected retinoblastoma cells (1 × 10^4^ per well) were seeded into 96-well plate (Corning, New York, NY, U.S.A.) and maintained at 37°C in humidified incubator for 24, 48, 72 and 96 h. Whereafter, 10 μl CCK-8 solution was added into each well and the retinoblastoma cells were maintained at 37°C for 1 h. The absorbance at 450 nm was detected by using a Microplate Reader (Bio-Rad, Hercules, CA, U.S.A.).

### Transwell migration and invasion assays

The 24-well transwell chambers (8-μm pore size; Corning, Franklin Lakes, NJ, U.S.A.) were used to conduct transwell migration and invasion assays. Briefly, 1 × 10^5^ retinoblastoma cells at 200 μl FBS-free RPMI-1640 medium were seeded into the upper chambers. Then, 500 μl RPMI-1640 medium with 20% FBS was added into bottom chambers. After incubation at 37°C for 24 h, the retinoblastoma cells on the upper chambers were removed, and the retinoblastoma cells on the lower chambers were fixed by 4% paraformaldehyde for 1 h and stained with 1% Crystal Violet for 15 min. Finally, the number of cells was counted under an inverted microscopy in five randomly chosen fields. The transwell invasion assay was performed similarly with transwell migration assay, except that chambers were pre-coated with Matrigel Basement Membrane Matrix (BD Biosciences, San Jose, CA, U.S.A.).

### Statistical analysis

All statistical analyses were performed using SPSS 17.0 (Chicago, IL, U.S.A.). The significance of the differences between two independent groups was estimated using Student’s *t* test. Correlations between FEZF1-AS1 expression and each clinicopathological parameter were evaluated using chi-square test. The disease-free survival (DFS) rate was calculated from the date of surgery to the date of progression. Survival curves were drawn by Kaplan–Meier method and estimated by log-rank test. Univariate and multivariate Cox proportional-hazard models were used for analyzing variables on DFS. *P*<0.05 was considered to indicate a statistically significant difference.

## Results

### FEZF1-AS1 expression is up-regulated in retinoblastoma tissues and cells

**Figure 1:**
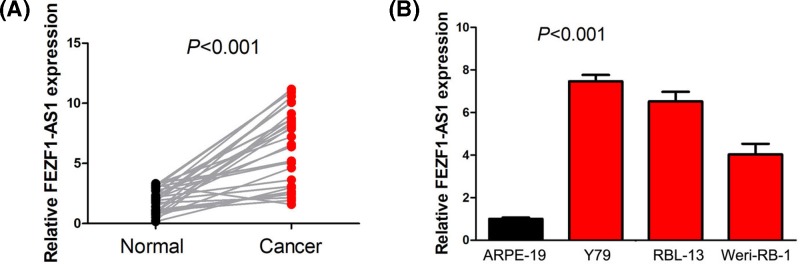
FEZF1-AS1 expression is up-regulated in retinoblastoma tissues and cells (**A**) Retinoblastoma tissue specimens exhibited higher FEZF1-AS1 expression than adjacent normal retina tissue specimens. (**B**) FEZF1-AS1 expression was obviously increased in human retinoblastoma cell lines (ERI-RB-1, Y79 and RBL-13) compared with human retinal pigment epithelial cell line (ARPE-19).

To investigate the expression pattern of FEZF1-AS1 in retinoblastoma, we first detected FEZF1-AS1 expression in retinoblastoma and adjacent normal retina tissue specimens. The results suggested that retinoblastoma tissue specimens exhibited higher FEZF1-AS1 expression than adjacent normal retina tissue specimens (Figure 1A). Furthermore, we detected levels of FEZF1-AS1 expression in human retinoblastoma cell lines (Y79, RBL-13 and Weri-RB-1) and human retinal pigment epithelial cell line (ARPE-19), and found FEZF1-AS1 expression was obviously increased in human retinoblastoma cell lines compared with human retinal pigment epithelial cell line (Figure 1B).

### FEZF1-AS1 overexpression is associated with the malignant status of retinoblastoma patients

For exploring the clinical significance of FEZF1-AS1 in retinoblastoma patients, all retinoblastoma patients were divided into two groups (high FEZF1-AS1 expression group and low FEZF1-AS1 expression group) according to the median FEZF1-AS1 expression level. Then, we analyzed the relationship between FEZF1-AS1 expression and the clinicopathological features of retinoblastoma patients, and found high FEZF1-AS1 expression was significantly correlated with present choroidal invasion and optic nerve invasion (Table 1). However, FEZF1-AS1 expression had no statistical correlation with age, gender, laterality and pathologic grade in retinoblastoma patients (Table 1).

**Table 1:** Correalations between FEZF1-AS1 and clinicopathological features in retinoblastoma

Features	*n*	High FEZF1-AS1 expression	Low FEZF1-AS1 expression	*P*
Age (years)				
≤2	31	15	16	0.796
>2	29	15	14	
Gender				
Male	35	15	20	0.190
Female	25	15	10	
Choroidal invasion				
No	36	11	25	<0.001
Yes	24	19	5	
Optic nerve invasion				
No	37	13	24	0.003
Yes	23	17	6	
Laterality				
Unilateral	43	19	24	0.152
Bilateral	17	11	6	
Pathologic grade				
Well differentiated	21	11	10	0.787
Poorly differentiated	39	19	20	

### FEZF1-AS1 overexpression is associated with unfavorable prognosis in retinoblastoma patients

**Figure 2:**
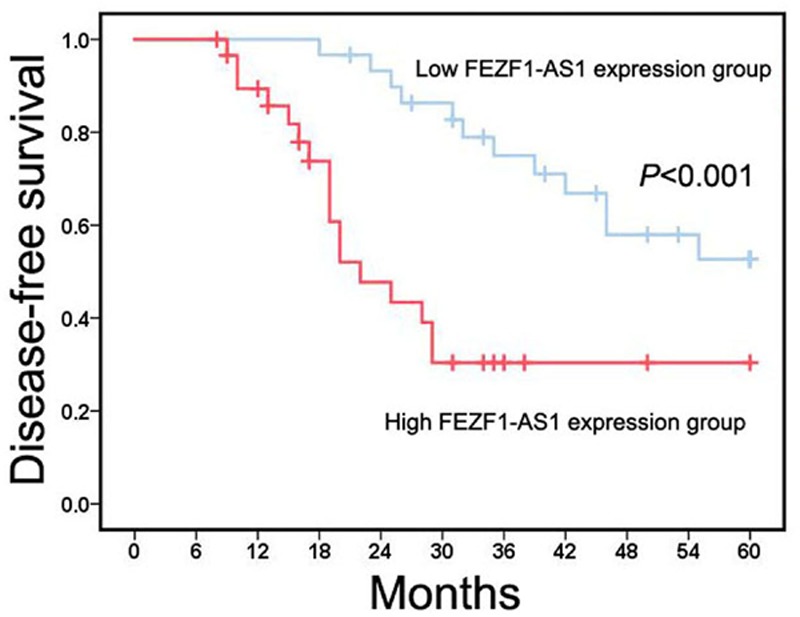
FEZF1-AS1 overexpression is associated with unfavorable prognosis in retinoblastoma patients The relationship between FEZF1-AS1 expression and DFS was evaluated through Kaplan–Meier method and log-rank test.

In order to estimate the prognostic value of FEZF1-AS1 in retinoblastoma patients, we analyzed the relationship between FEZF1-AS1 expression and DFS. We found that retinoblastoma patients in high FEZF1-AS1 expression group had obviously shorter DFS compared with retinoblastoma patients in low FEZF1-AS1 expression group (Figure 2). Furthermore, we estimated that potential prognostic factors for DFS in retinoblastoma patients through univariate and multivariate Cox proportional-hazard models. The results of univariate Cox proportional-hazard models showed choroidal invasion, optic nerve invasion and FEZF1-AS1 expression were prognostic factors in retinoblastoma patients (Table 2). Moreover, multivariate Cox proportional-hazard models indicated high FEZF1-AS1 expression was an independent unfavorable prognostic factor for DFS in retinoblastoma patients (Table 2).

**Table 2:** Univariate and multivariate Cox regression of prognostic factors for DFS in retinoblastoma patients

Parameter	Univariate analysis			Multivariate analysis		
	HR	95%CI	*P*	HR	95%CI	*P*
Age (years)						
≤2 vs. >2	1.072	0.517–2.224	0.851			
Gender						
Male vs. Female	1.059	0.506–2.218	0.879			
Choroidal invasion						
No vs. Yes	3.218	1.476–7.018	0.003	1.464	0.543–3.946	0.452
Optic nerve invasion						
No vs. Yes	3.150	1.493–6.646	0.003	2.315	1.024–5.230	0.044
Laterality						
Unilateral vs. Bilateral	1.972	0.918–4.235	0.082			
Pathologic grade						
Well differentiated vs. Poorly differentiated	0.562	0.269–1.176	0.126			
FEZF1-AS1						
Low vs. High	3.703	1.703–8.052	0.001	2.618	1.008–6.802	0.048

Abbreviations: HR, hazard ratio; 95%CI, 95% confidence interval.

### Silencing FEZF1-AS1 expression inhibits retinoblastoma cell proliferation, invasion and migration

**Figure 3:**
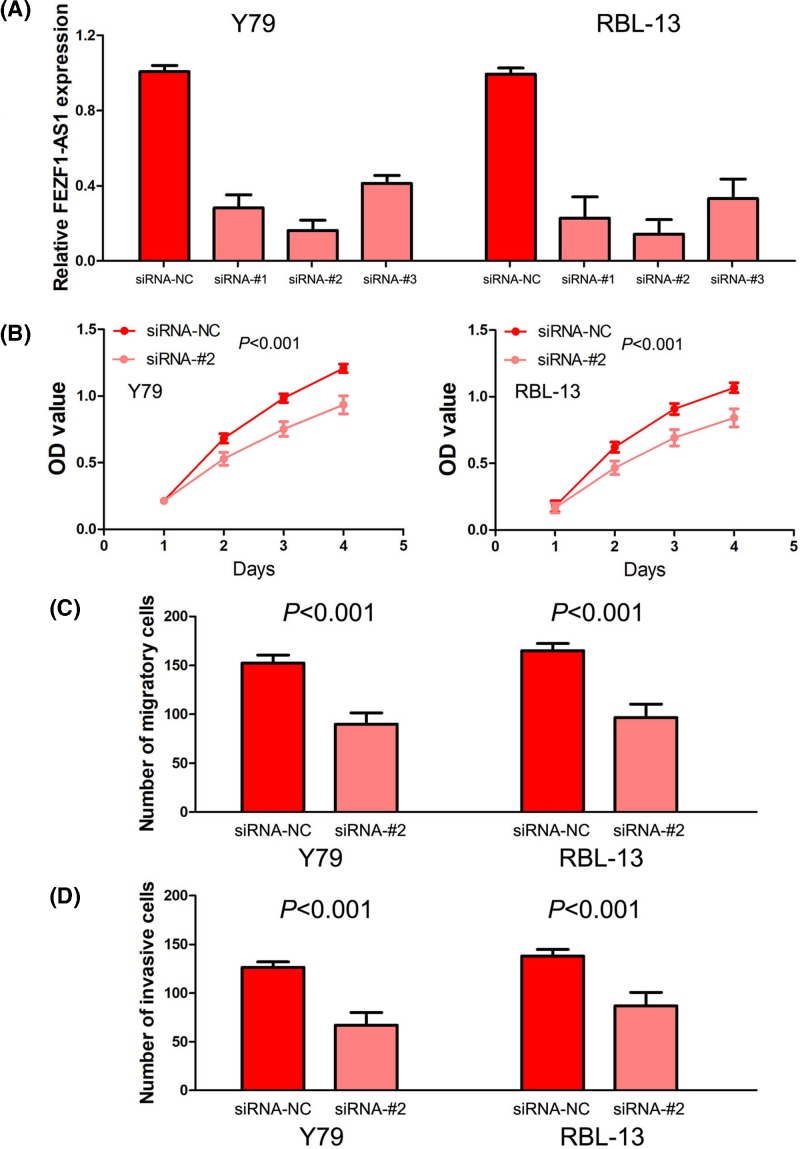
Silencing FEZF1-AS1 expression inhibits retinoblastoma cell proliferation, invasion and migration (**A**) The qRT-PCR was utilized to confirm the transfection efficiencies of FEZF1-AS1-siRNAs in retinoblastoma cells. (**B**) The effects of FEZF1-AS1 on retinoblastoma cell proliferation were evaluated by CCK-8 assay. (**C,D**) Transwell migration and invasion assays were used to assess the influences of FEZF1-AS1 on retinoblastoma cell invasion and migration.

For exploring the effects of FEZF1-AS1 on cell’s biological behavior, we performed loss-of-function study in retinoblastoma cells through siRNA transfection. After transfection with FEZF1-AS1-siRNA#1, FEZF1-AS1-siRNA#2, and FEZF1-AS1-siRNA#3, we found FEZF1-AS1-siRNA#2 was the most effective siRNA and chosen for following experiments *in vitro* (Figure 3A). The effects of FEZF1-AS1 on retinoblastoma cell proliferation were evaluated by CCK-8 assay. The results showed silencing FEZF1-AS1 expression remarkably suppressed cell proliferation ability of retinoblastoma cells (Figure 3B). In addition, transwell migration and invasion assays were used to assess the influence of FEZF1-AS1 on retinoblastoma cell invasion and migration. We found silencing FEZF1-AS1 expression dramatically repressed retinoblastoma cell invasion and migration abilities of retinoblastoma cells (Figure 3C,D).

## Discussion

In recent years, increasing studies suggested that FEZF1-AS1 was significantly overexpressed in many types of human cancers, such as lung cancer [18–21], breast cancer [22], liver cancer [23,24], gastric cancer [25–27], colorectal cancer [28,29], pancreatic cancer [30,31], ovarian cancer [32], cervical cancer [33], osteosarcoma [34], nasopharyngeal carcinoma [35] and multiple myeloma [36]. However, the expression of FEZF1-AS1 was still unclear in retinoblastoma. In our study, we first detected FEZF1-AS1 expression in retinoblastoma tissue specimens and cells, and found levels of FEZF1-AS1 expression were elevated in retinoblastoma tissue specimens and cell lines compared with adjacent normal retina tissue specimens and human retinal pigment epithelial cell line, respectively. Furthermore, we analyzed the relationship between FEZF1-AS1 expression and the clinicopathological features for exploring the clinical significance of FEZF1-AS1 in retinoblastoma patients. The correlation analysis indicated that high FEZF1-AS1 expression was significantly correlated with present choroidal invasion and optic nerve invasion. In lung cancer patients, three clinical studies congruously showed that high FEZF1-AS1 expression was correlated with advanced clinical stage [18,19,21]. Besides, Zhang et al. [22] found high FEZF1-AS1 expression was related with advanced TNM stage, lymphatic metastasis, distant metastasis, positive HER2 expression and ER expression in breast cancer patients. In addition, Wang et al. [24] reported FEZF1-AS1 overexpression was associated with large tumor size, and late TNM stage and present venous invasion in patients with hepatocellular carcinoma. In gastric cancer patients, Liu et al. [25] and Wu et al. [27] both showed high FEZF1-AS1 expression was often observed in patients with advanced clinical stage, large tumor size or poor tumor grade. Additionally, the similar clinical significance of FEZF1-AS1 was also observed in colorectal cancer [28,29], pancreatic cancer [30,31], cervical cancer [33], osteosarcoma [34] and nasopharyngeal carcinoma [35].

High FEZF1-AS1 expression in tumor tissues has been suggested to be an independent unfavorable prognostic predictor in several types of tumors. In lung cancer, high FEZF1-AS1 expression was associated with poor overall survival, and acted as an independent prognostic factor for overall survival in lung adenocarcinoma patients [20,21]. Similar results in colorectal cancer patients were reported, these studies indicated that FEZF1-AS1 expression was negatively correlated with overall survival time, and FEZF1-AS1 overexpression served as an independent poor prognostic factor for overall survival [28,29]. In cervical cancer patients, Zhang and Li [33] suggested high-level expression of FEZF1-AS1 was correlated with poor prognosis, and was considered an unfavorable independent prognostic biomarker for overall survival. In addition, there was negative correlation between FEZF1-AS1 expression and clinical outcome in breast cancer [22], liver cancer [23,24], gastric cancer [25,27], pancreatic cancer [30,31], ovarian cancer [32], osteosarcoma [34] and nasopharyngeal carcinoma [35]. The prognostic value of FEZF1-AS1 expression was still unknown in retinoblastoma patients. In our research, we found retinoblastoma patients in high FEZF1-AS1 expression group had obviously shorter DFS compared with retinoblastoma patients in low FEZF1-AS1 expression group, and high FEZF1-AS1 expression was an independent unfavorable prognostic factor for DFS in retinoblastoma patients, which was similar to the prognostic value of FEZF1-AS1 expression in other types of human cancers.

Based on the above clinical study about the role of FEZF1-AS1 expression in retinoblastoma patients, we guessed that FEZF1-AS1 functions as an oncogenic lncRNA to regulate biological behavior in retinoblastoma. Thus, we performed loss-of-function study in retinoblastoma cells, and found silencing FEZF1-AS1 expression inhibited retinoblastoma cell proliferation, invasion and migration. In addition, FEZF1-AS1 was suggested to promote tumor cell proliferation, migration and invasion through activating Wnt/β-catenin signaling pathway in lung cancer [18], gastric cancer [27] and nasopharyngeal carcinoma [35]. Besides, FEZF1-AS1 acted as sponge toward miR-30a/Nanog [22], miR-107/ZNF312B [30], miR-142/HIF-1α [31], miR-133a/EGFR [31], miR-4443/NUPR1 [34] and miR-610/Akt3 [36]. Regrettably, the limit of our study lacked the molecular mechanism of FEZF1-AS1 in retinoblastoma.

In conclusion, FEZF1-AS1 was up-regulated in retinoblastoma tissues and cells, and correlated with aggressive phenotypes and poor clinical outcome in retinoblastoma patients. Silencing FEZF1-AS1 expression inhibits retinoblastoma cell proliferation, invasion and migration.

## Abbreviations

CCK-8: cell counting kit-8
DFS: disease-free survival
FBS: fetal bovine serum
FEZF1-AS1: FEZF1 antisense RNA 1
lncRNA: long non-coding RNA
RPMI: Roswell Park Memorial Institute
TNM: tumor node metastasis
